# Dr. L. P. Yandall

**Published:** 1881-02

**Authors:** 


DR. L. P. YANDALL has resigned his position as senior editor of *The Louisville Medical News*. The junior editor, Dr. Cowling, will have exclusive control of the Journal in future. We wish him and his publication continued success.

